# TNF signaling inhibition in the CNS: implications for normal brain function and neurodegenerative disease

**DOI:** 10.1186/1742-2094-5-45

**Published:** 2008-10-17

**Authors:** Melissa K McCoy, Malú G Tansey

**Affiliations:** 1 Department of Physiology, The University of Texas Southwestern Medical Center at Dallas, Dallas, TX 75390-9040, USA

## Abstract

The role of tumor necrosis factor (TNF) as an immune mediator has long been appreciated but its function in the brain is still unclear. TNF receptor 1 (TNFR1) is expressed in most cell types, and can be activated by binding of either soluble TNF (solTNF) or transmembrane TNF (tmTNF), with a preference for solTNF; whereas TNFR2 is expressed primarily by microglia and endothelial cells and is preferentially activated by tmTNF. Elevation of solTNF is a hallmark of acute and chronic neuroinflammation as well as a number of neurodegenerative conditions including ischemic stroke, Alzheimer's (AD), Parkinson's (PD), amyotrophic lateral sclerosis (ALS), and multiple sclerosis (MS). The presence of this potent inflammatory factor at sites of injury implicates it as a mediator of neuronal damage and disease pathogenesis, making TNF an attractive target for therapeutic development to treat acute and chronic neurodegenerative conditions. However, new and old observations from animal models and clinical trials reviewed here suggest solTNF and tmTNF exert different functions under normal and pathological conditions in the CNS. A potential role for TNF in synaptic scaling and hippocampal neurogenesis demonstrated by recent studies suggest additional in-depth mechanistic studies are warranted to delineate the distinct functions of the two TNF ligands in different parts of the brain prior to large-scale development of anti-TNF therapies in the CNS. If inactivation of TNF-dependent inflammation in the brain is warranted by additional pre-clinical studies, selective targeting of TNFR1-mediated signaling while sparing TNFR2 activation may lessen adverse effects of anti-TNF therapies in the CNS.

## Introduction

The potent pro-inflammatory cytokine Tumor necrosis factor (TNF) is a member of the TNF superfamily of ligands, many of which promote inflammatory signaling [1-3]. TNF is synthesized as a monomeric type-2 transmembrane protein (tmTNF) that is inserted into the membrane as a homotrimer and cleaved by the matrix metalloprotease TNF alpha converting enzyme (TACE; ADAM17) to a 51 kDa soluble circulating trimer (solTNF); both tmTNF and solTNF are biologically active (reviewed in [4-6]), and can be synthesized in the central nervous system (CNS) by microglia, astrocytes, and some populations of neurons [7-9]. The balance between tmTNF and solTNF signaling is influenced by cell type, activation status of the cell, the stimulus eliciting TNF production, TACE activity, and expression of endogenous TACE inhibitors leading to divergent TNF-mediated effects on cellular viability [10,11].

### TNF receptors

TNF receptor 1 (TNFR1, Tnfrsf1a) and TNF receptor 2 (TNFR2, Tnfrsf1b) are membrane glycoprotein receptors that specifically bind TNF and homotrimers of lymphotoxin A, but the two receptors differ in their expression profiles, ligand affinity, cytoplasmic tail structure, and downstream signaling pathway activation (reviewed in [12]). Signaling of TNF through TNFRs requires that receptors preassemble on the cell membrane as trimers prior to ligand binding, this trimerization occurs through the intracellular cytoplasmic tail of the receptors and trimers are composed of like receptors due to the divergent sequence of their intracellular domains [13,14]. TNFR1 is expressed in most cell types, and can be activated by binding of either solTNF or tmTNF, with a preference for solTNF; whereas TNFR2 is expressed primarily by cells of the immune system (including microglia) and by endothelial cells, and is preferentially activated by tmTNF [15,16].

### Intracellular signaling pathways activated by TNF receptors

TNF signaling through TNFR1 and TNFR2 can elicit a variety of cellular responses depending on many factors including the metabolic state of the cell and the adaptor proteins present in the cell. These differences then influence the activation of a number of intracellular signaling pathways including nuclear factor kappa-B (NF-κB), p38, c-jun N-terminal kinase (JNK), and the ceramide/sphingomyelinase signaling pathway, resulting in a number of responses including inflammation, proliferation, cell migration, apoptosis, and necrosis [17-20].

### TNFR1-mediated signaling

SolTNF signaling is thought to elicit its biological effects primarily through TNFR1 activation. TNFR1 contains a cytoplasmic death domain (DD) characteristic of many members of the TNF superfamily that permits the assembly of the TNFR1 signaling complex through the dissociation of silencer of death domains (SODD) and subsequent binding of TNF receptor associated death domain (TRADD) [21,22]. Binding of TRADD then allows the recruitment of other adaptor proteins including receptor interacting protein (RIP) and TNF receptor associated factor 2 (TRAF2) [23-26]. This complex then leads to RIP-dependent activation of NFκB signaling to initiate pro-survival signaling, cellular proliferation, and cytokine production. This membrane associated complex of ligand-engaged TNFR1 with TRADD, TRAF2, and RIP also recruits cellular inhibitor of apoptosis proteins 1 and 2 (cIAP 1,2) resulting in activation of ERK, JNK, p38 MAP kinase, and ceramide/sphingomyelinase pathways [27-30]. The kinetics of JNK activation is particularly important in determining the functional outcome of a TNF signal. Acute, transient TNF-induced JNK activation, which is cytoprotective, results from TAK1-dependent phosphorylation [31], whereas sustained JNK activation leading to caspase-dependent apoptosis depends upon JNK phosphorylation by apoptosis signal-regulating kinase 1 (ASK1) [32]. Knock-in mice expressing mutant TNF that is no longer a substrate for TACE cleavage (and therefore remains membrane bound) have demonstrated solTNF to be the primary mediator of TNF-dependent inflammatory responses. In these transgenic mice, lymphoid organ development is relatively normal (with the exception of primary B cell follicles), but the initiation of autoimmune pathology is severely compromised [33].

In addition to TNFR1-induced activation of stress-induced kinase signaling in contributing to apoptotic signaling, TNFR1 can be internalized after TNF binding and this leads to dissociation of the TRADD/TRAF2/RIP complex and association of Fas-associated DD (FADD), recruitment of pro-caspase 8, and formation of the death-inducing signaling complex (DISC), triggering activation of the executioner caspases through the extrinsic apoptosis pathway [34,35]. Caspase-8, combined with its ability to induce apoptosis through the extrinsic pathway, also triggers the intrinsic apoptosis pathway by cleaving the pro-apoptotic Bcl-2 family members Bax and Bid to initiate mitochondrial-induced apoptosis [36-38]. The complexity of TNFR1-mediated signaling is what allows many divergent (ie, proliferation, activation, apoptosis) outcomes to occur as a result of TNF signaling and contributes to the difficulties inherent with and the side effects resulting from broad TNF signaling inhibition, particularly in areas including the CNS where TNF signaling has been demonstrated to be functionally important for homeostatic process as discussed in the sections below.

### TNFR2-mediated signaling

TNFR2 is restricted to expression in endothelial, hematopoetic, and some neuronal populations and displays preferential binding to tmTNF, both of these characteristic are likely to result in fewer biological effects compared to those mediated by TNFR1-dependent signaling [16]. Signaling through TNFR2 activates inflammatory and pro-survival signaling pathways through recruitment of TRAF1 and TRAF2 adaptor proteins and subsequent activation of cIAPs and the NF-κB pathway [39-41]. TNFR2 has also been shown to activate phosphatidylinositol 3-kinase-dependent signaling to promote neuron survival [42,43]. TNFR2 can promote TNFR1 signaling by enhancing the association between solTNF and TNFR1 via a ligand passing mechanism, and it has been suggested that this ligand passing is the primary contribution of TNFR2 to TNF-mediated signaling in contrast to direct signaling pathway activation through adaptor protein association with the intracellular domain of TNFR2 [44]. TNFR2 does not contain a DD, and thus, unlike signaling through TNFR1, TNFR2 activation does not directly lead to caspase activation. Overall, TNFR2 activation is believed to initiate primarily pro-inflammatory and pro-survival signaling.

### Reverse signaling through transmembrane TNF

Upon receptor binding, tmTNF has been demonstrated to initiate intracellular signaling in the tmTNF-expressing cell [20,45]. This signaling is mediated, at least in part, through casein kinase 1 phosphorylation of the cytoplamsic tail of TNFR2 resulting in increased intracellular calcium levels and activation of p38 and MAP kinase pathways [46-48]. Another type of reverse signaling though tmTNF is possible; after release of solTNF by TACE, the TNF intracellular domain (TNF-ICD) can be released into the cell through regulated intramembrane proteolysis by signal peptide peptidase-like proteases where it is trafficked to the nucleus by virtue of a nuclear localization signal resulting in increased production of pro-inflammatory cytokines [49-51]. A detailed understanding of the signaling that occurs through tmTNF is still underway and the *in vivo *significance of these reverse signaling pathways remains to be established.

### TNF signaling in the brain

TNF signaling has been shown to have several important functions within the CNS [52] including injury-mediated microglial and astrocyte activation, and regulation of blood brain barrier permeability, febrile responses, glutamatergic transmission, and synaptic plasticity and scaling [53-58]. Excitatory synapse scaling resulting from activity blockade has been shown to be mediated by TNF-dependent increases in AMPA receptors at the cell surface and decreases in GABA_A _receptor cell surface expression, suggesting that TNF may control synaptic strength at excitatory synapses by increasing excitatory synaptic transmission and reducing inhibitory transmission [57,59,60]. TNF was demonstrated to increase the mean frequency of miniature excitatory postsynaptic currents (mEPSCs) [57,59]. In addition TNF has been shown to increase mEPSC amplitude and decrease mIPSC (miniature inhibitory postsynaptic currents) amplitude [60]. Pharmacologically, TNFR or anti-TNF antibody treatment could prevent basal and tetrototoxin-induced increases in surface expression of AMPA receptors as well as increases in mEPSC amplitude and decreases in mIPSC amplitude, further supporting solTNF as an important mediator of synaptic scaling [57,59]. Synaptic scaling was also examined in hippocampal cultures from TNF deficient mice and chronic activity blockade did not result in increased mEPSC amplitude which is seen in wildtype cultures confirming the TNF dependence of this increase [59].

The function of TNF signaling through TNFR1 and TNFR2 in the hippocampus and cortex has not been fully elucidated. On the one hand, TNF appears to regulate hippocampal neuronal development as TNF-deficient mice display accelerated maturation of the dentate gyrus region and smaller dendritic trees in the hippocampus [61]. In contrast, TNF can potentiate excitotoxicity by two mechanisms. In combination with sub-threshold glutamate levels, TNF can potentiate glutamate excitotoxicity directly through activation of glutamate-NMDA receptors [62] and localization of AMPA receptors to synapses [63-65], and indirectly by inhibiting glial glutamate transporters on astrocytes [66]. However, findings that suggest TNFR2 signaling protects against excitotoxicity have also been reported. Specifically, cortical neurons from mice that overexpress TNF in the CNS were protected from glutamate toxicity as were wildtype and TNFR1-deficient mice pretreated with TNF or agonistic TNFR2 antibodies; consistent with those findings, neurons derived from TNFR2-deficient mice were susceptible to both TNF and glutamate-induced death [43]. In these studies, protein kinase B/Akt dependent activation of NFκB was necessary for neuronal survival after glutamate exposure [43].

Genetic manipulation of TNF or TNF receptors in mouse models of disease have provided valuable insight into the biological roles of TNF in the CNS [52]. Elevated levels of TNF are evident in a large number of neurological disorders including ischemia [67,68], traumatic brain injury [69], multiple sclerosis [70-74], Alzheimer's disease [75-77], and Parkinson's disease [78-83], but whether TNF signaling actively contributes to or limits neuronal injury in these disorders has yet to be established. A number of pre-clinical and clinical studies in various disease models and in chronic neurodegenerative conditions suggest that targeting TNF action in the brain may be an attractive disease-modifying strategy to slow progression or attenuate severity of the disease and are discussed in sections below.

### TNF inhibitors

#### Endogenous TNF inhibitors

A number of endogenous mechanisms limit TNF activity during inflammatory responses as part of resolution of those responses (reviewed in [84]). Identified endogenous inhibitors of TNF include prostaglandins and cyclic AMP which limit TNF production and glucocorticoids which are produced when TNF levels are high by activation of the hypothalamus pituitary adrenal axis to inhibit TNF production [84-88]. Anti-inflammatory cytokines including IL-10, IL-13 and IL-4 can inhibit TNF production [89-92]. TACE cleaves tmTNF to generate solTNF and it can also cleave TNF receptors to generate solTNFRs which can bind solTNF in the circulation. In fact, soluble forms of TNF receptors were first identified in urine and their discovery lead to the development of anti-TNF antibody-based biologics [93-96]. Efferent activity of the vagus nerve has also been shown to inhibit TNF production through cholinergic activation of muscarinic receptors [97-99].

#### Biologics

**Figure 1:**
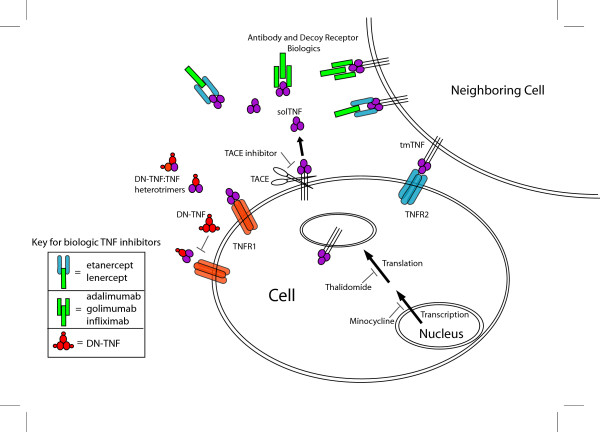
**Schematic of TNF inhibitors and their mode of action**. Receptor (i.e. etanercept, lenercept) and antibody based (i.e. infliximab, adalimumab, golimumab) anti-TNF biologics inhibit both solTNF and tmTNF. TNF variants (DN-TNFs) exchange with native solTNF monomers to form heterotrimers with drastically reduced abilities to bind TNF receptors, making them selective for solTNF signaling inhibition. Small molecule inhibitors of TNF signaling include minocycline which decreases TNF synthesis, thalidomide which enhances degradation of TNF mRNA, and TACE inhibitors which prevent TACE induced release of solTNF.

A number of protein-based TNF inhibitors which we will refer to as *biologics *are available or currently under development (Table 1), and a subset of these have been approved for use in the treatment of peripheral autoimmune disorders including rheumatoid and juvenile arthritis, ankylosing spondylitis, and Crohn's disease. Non-selective biologic inhibitors include, but are not limited to, infliximab, etanercept, and adalimumab, all of which bind to soluble and tmTNF and prevent binding to TNF receptors (Figure 1). Infliximab is a chimeric bivalent IgG1 monoclonal antibody composed of a human constant region and murine variable regions, adalimumab is a humanized bivalent mouse IgG1 monoclonal antibody, and etanercept is a fusion protein comprised of human IgG fused to a dimer of the extracellular regions of TNFR2. A number of important differences exist between TNF inhibitors in their mode of action and specificity that are worth noting (Table 1). Infliximab and adalimumab, by virtue of being IgGI antibodies, can activate complement and bind Fc Receptor and they also can bind both monomeric and trimeric solTNF whereas etanercept, like TNFR2, only binds TNF trimers, however all three inhibitors bind tmTNF and therefore inhibit signaling through both TNF receptors [100-103]. Another difference between etanercept and infliximab and adalimumab is the ability of etanercept to neutralize lymphotoxin, a property that is shared by its parent receptor TNFR2 [100,104]. In addition to these approved inhibitors several similar antibody and receptor based TNF inhibitors are currently or were previously under clinical investigation including lenercept, a dimeric TNFR1 receptor extracellular domain fused to a human IgG1 heavy chain fragment, and the fully human monoclonal antibody golimumab (Table 1).

**Table 1:** Selected TNF inhibitors.

**Inhibitor (Trade name)**	**Class**	**Description/Mode of Action**	**Specificity**	**Developer and Stage**	**References**
Adalimumab (Humira)	Monoclonal antibody	Humanized bivalent mouse IgG1 monoclonal antibody; binds to ligands	solTNF, tmTNF	Abbott FDA approved 2005	[100-103,179,180]
Apratastat	Small molecule	Dual TACE and MMP inhibitor	MMPs, TACE	Wyeth Phase II terminated 2006	[121]
BMS-561392	Small molecule	Specific TACE inhibitor	TACE	Bristol-Myers Squibb Phase II	[121]
Certolizumab (Cimzia)	Monoclonal antibody	PEGylated antibody fragment	solTNF, tmTNF	UCB FDA approved 2008	[182]
CYT007-TNFQb	Vaccine	Anti-TNF vaccine	TNF	Cytos Biotechnology Phase I-IIa	[181]
DN-TNF (XPro1595)	Inactive TNF variant	TNF monomers engineered with point mutations to disrupt binding to TNFRs; exchange with solTNF to form dominant-negative heterotrimers; tmTNF-sparing	solTNF	Xencor Preclinical	[105,106]
ESBA105	Antibody fragment	Single-chain antibody fragment directed against TNF	TNF	Esbatech (Zurich) Preclinical	[181]
Etanercept (Enbrel)	Receptor biologic	Human IgG fused to a dimer of the extracellular regions of TNFR2; binds to ligands	solTNF, tmTNF, Lympotoxin A	Amgen; Wyeth; Takeda FDA approved 1998	[179,180]
Golimumab (CNTO148)	Monoclonal antibody	Human monoclonal antibody; binds to ligands	solTNF, tmTNF	Centocor, Schering-Plough Phase III	[101]
GW333	Small molecule	Dual TACE and MMP inhibitor	MMPs, TACE	GlaxoSmithKline Preclinical	[121]
Infliximab (Remicade)	Monoclonal antibody	Murine-human Chimeric bivalent IgG1 monoclonal antibody; binds to ligands	solTNF, tmTNF	Centocor Schering-Plough FDA approved 1998	[179,180]
Lenercept	Receptor biologic	Dimeric TNFR1 receptor extracellular domain fused to a human IgG1heavy chain fragment; binds to ligands	solTNF, tmTNF, Lympotoxin A	Roche Phase III terminated	[101,131,183]
Minocycline	Small molecule	Broad-spectrum tetracycline antibiotic; inhibits synthesis of TNF and other inflammatory mediators	solTNF, tmTNF, MMPs, COX-2, prostaglandin E2	Off patent	[110-117]
tgAAC94	Gene therapy	AAV vectors containing the TNFR2:Fc fusion	solTNF, tmTNF, Lympotoxin A	Targeted Genetics Phase I-II	[184]
Thalidomide	Small molecule	Immunomodulatory drug; increases degradation of mRNA of a number of inflammatory genes	TNF, COX-2, IL-1β, TGF-β, IL-12 and IL-6.	Celgene Corporation FDA approved 1998	[118,119]

Abbreviations: solTNF, soluble tumor necrosis factor; tmTNF, transmembrane tumor necrosis factor; TACE, tumor necrosis factor alpha converting enzyme; MMP, matrix metalloprotease; DN-TNF, dominant-negative tumor necrosis factor; TNFR, tumor necrosis factor receptor; COX-2, cyclo-oxygenase-2; IL1-β, interleukin-1beta; TGF-β, transforming growth factor beta; IL-12, interleukin-12; IL-6, interleukin-6.

A new class of anti-TNF biologic with a dominant-negative (DN) mechanism of action has emerged and was developed using structure-based computational design leading to the generation of TNF variants referred to as DN-TNFs which are able to exchange with native soluble TNF monomers to form heterotrimers (Figure 1) with drastically reduced abilities to bind TNF receptors [105,106]. Due to this novel mechanism of action, DN-TNFs selectively inhibit solTNF and do not inhibit tmTNF signaling [105,107]. The ability of DN-TNFs to be tmTNF-sparing and solTNF-selective may be ideal in clinical conditions where solTNF signaling mediates pathology, and/or to ensure that the role of tmTNF in immune function is not compromised.

#### Small molecule TNF inhibition

A small molecule TNF inhibitor that has been described is composed of trifluoromethylphenyl indole and dimethyl chromone moieties linked by a dimethylamine spacer [108]. This small molecule inhibitor binds to trimerized TNF and accelerates subunit dissociation to lead to a TNF dimer/small molecule complex that can not bind to and activate TNFR1 [108].

#### Immunomodulatory drugs with TNF inhibitory action

Less specific inhibitors of TNF include minocycline and thalidomide which have been used in the treatment of inflammatory conditions. Minocycline, a broad-spectrum tetracycline antibiotic, has been shown to have both bacteriostatic and anti-inflammatory actions [109]. Minocycline not only decreases TNF synthesis, but also inhibits matrix metalloproteases, reduces COX-2 activity and prostaglandin E2 production, and attenuates apoptosis [110-117]. Thalidomide is an immunomodulatory drug which means it can attenuate a variety of cytokines and immune cell-mediated responses. Thalidomide inhibits TNF through enhanced degradation of TNF mRNA [118,119], however is can also alter the expression of COX-2, IL-1β, TGF-β, IL-12 and IL-6 to affect immune cell regulation and migration separate from its TNF-dependent effects (reviewed in [118]).

#### TACE inhibitors

Another method by which inhibition of soluble TNF production can be achieved is by inhibition of TACE, the protease that cleaves and converts tmTNF into its circulating form. TACE, a member of the ADAM (A Disintegrin And Metalloproteinase protein) family of matrix metalloproteases, is the primary enzyme responsible for TNF shedding *in vivo *[120]. Unlike what has been possible to achieve with anti-TNF biologics, development of orally available brain-permeant TACE inhibitors has been successful. TACE inhibitors are in various stages of clinical development, but there are limitations with current inhibitors including non-specific inhibition of other matrix metalloproteases, and inhibition of other TACE substrates; both properties may lead to undesired side-effects. Current TACE inhibitors include two small-molecule dual TACE and MMP inhibitors, GW333 and apratastat (TMI-005), and a more specific TACE inhibitor BMS-561392 (reviewed in [121]). Development of apratastat for the treatment of rheumatoid arthritis was terminated after phase II clinical trials due to lack of efficacy [122]. Other TACE inhibitors including BMS-561392 have been delayed from clinical trials due to the potential risk of hepatoxicity resulting from the accumulation of tmTNF and the inhibition of TNFR1 and TNFR2 shedding which was demonstrated in rats treated with TACE inhibitors (reviewed in [121]).

### TNF in multiple sclerosis (MS)

Evidence that implicates TNF in the underlying pathology of multiple sclerosis (MS) includes: the observation that at autopsy MS patients have elevated TNF levels at the site of active MS lesions [70]; reports that CSF and serum TNF levels in individuals with MS are elevated compared to unaffected individuals and TNF levels correlate to the severity of the lesions [72,123,124]; and evidence that peripheral blood mononuclear cells from MS patients just prior to symptom exacerbation have increased TNF secretion after stimulation compared to cells form the same patients during remission [73,125]. These characteristics of TNF involvement are shared with peripheral autoimmune diseases. Based on these strong clinical parameters implicating TNF signaling in contributing to MS disease severity, the effects of manipulation of the TNF pathway were investigated in mouse models of MS. Specifically, overexpression of TNF leads to demyelinating disease and neutralization of TNF with anti-TNF antibodies or receptor fusion proteins is protective in experimental autoimmune encephalomyelitis (EAE) transgenic mouse models [126-130]. Logically, based upon promising results in these animal models, lenercept, a dimeric TNFR1 receptor extracellular domain fused to a human IgG1heavy chain fragment, was evaluated in MS patients. Unfortunately, a phase II randomized, multi-center, placebo-controlled study had to be halted due to a dose-dependent increase in MS attack frequency and a trend towards increased attack duration and severity [131]. Although the reasons for unanticipated failure were not immediately clear, the failed lenercept clinical trial served to prompt further investigations into the action of TNF in nerve myelination in animal models of MS and in transgenic mice with alterations in TNF and its receptors. Subsequently, TNFR1 signaling has been demonstrated to mediate nerve demyelination whereas TNFR2 signaling appears to be crucial for remyelination in the cuprizone mouse model of MS. Specifically, it was shown that the absence of TNF resulted in delayed remyelination, depletion of the oligodendrocyte progenitors, and reduction in mature astrocytes after cuprizone withdrawal with TNFR2 expression alone being sufficient to restore oligodendrocyte regeneration [132]. Interestingly, the importance of TNF signaling in mediating nerve demyelination was found to occur independently of the adaptive immune response as the Tg6074 line of TNF overexpressing mice, even when backcrossed to mice deficient in CD4 (to ablate CD4+ lympocytes), β2-microglobulin (MHC I restricted T lymphocytes), immunoglobulin μ (Ig+ lymphocytes), or Rag-1 (T Cell receptor and Ig positive lymphocytes), still develop primary demyelination to the same extent as mice with an intact adaptive immune response [133]. These important findings in pre-clinical models have clinical implications and suggest that inhibition of tmTNF/TNFR2 signaling by lenercept was the molecular basis for the worsening symptoms in MS patients. Experiments to block solTNF/TNFR1 signaling with the solTNF-selective DN-TNF inhibitors in the cuprizone model should yield further support for this hypothesis. Transgenic animal models in which tmTNF is expressed exclusively demonstrated that tmTNF, in the absence of solTNF, suppresses EAE disease onset and progression, while still maintaining the ability of TNF signaling to suppress autoimmune properties [134,135]. This ability of tmTNF to maintain immune functions such as self-tolerance and resistance to infection while limiting other TNF functions including primary demyelination and oligodendrocyte apoptosis [135] opens the possibility of selective inhibition of solTNF/TNFR1 signaling as a therapeutic strategy to prevent relapsing-remitting MS in patients afflicted with this chronic inflammatory disease of the nervous system.

### TNF in ischemia (stroke) models

There is conflicting evidence concerning the role of TNF in ischemia (stroke) models (reviewed in [136]). For example, TNF action has been inhibited in acute stroke through intraventricular infusion of TNFR1 decoy receptor or anti-TNF antibody, and through systemic administration of a TACE inhibitor and both approaches resulted in reduced ischemic injury [137-139]. However, hippocampal neurogenesis after ischemic injury resulting from stroke was abolished when animals were treated with anti-TNF antibodies two weeks after the ischemic event, suggesting that TNF signaling may be necessary for repair processes after an ischemic insult [140]. In addition, epileptic seizures resulting from focal cerebral ischemia worsened in mice deficient for TNF or TNF receptors, further suggesting that TNF signaling is important for hippocampal function following ischemic injury [141]. The mechanism of TNF-induced protection from epileptic development was investigated in TNF and TNF receptor transgenic mouse models. Specifically, astrocyte restricted overexpression of TNF or intrahippocampal injection of TNF was found to reduce susceptibility to kainate-induced seizure activity, whereas single TNFR2 or double TNFR1/TNFR2-deficient mice displayed prolonged seizure activity, implicating TNFR2 and tmTNF signaling in protection against seizure [142]. In summary, studies with TNF antibodies in stroke and seizure models raise the interesting possibility that TNF action through TNFR2 is important in hippocampal repair and neurogenesis, and suggest that use of anti-TNF drugs that do not spare TNFR2-dependent signaling may result in untoward effects in this brain region.

### TNF in Parkinson's disease (PD)

As in many other neurodegenerative diseases, TNF and solTNFR1 levels are elevated in cerebrospinal fluid and tissues of PD patients as well as in postmortem PD brains with the highest TNF levels present in areas that have the greatest loss of dopaminergic neurons [78-81]. Nevertheless, the importance of TNF signaling in contributing to DA neuron dysfunction and death has only recently been appreciated. In animal models of PD increased TNF mRNA and protein are detectable in the midbrain within hours of *in vivo *administration of a neurotoxic dopamine analog, 6-hydroxydopamine (6-OHDA) [143], the mitochondrial complex I inhibitor 1-methyl-4-phenyl-1,2,3,6-tetrahydropyridine (MPTP) [144-146], and the bacterial endotoxin lipopolysaccharide (LPS) [147]. In MPTP-treated non-human primates, serum TNF levels have been reported to remain elevated up to one year after a single MPTP injection [148]. Multiple studies indicate that TNF is highly toxic to dopaminergic neurons in both *in vitro *primary cultures [149-151] and *in vivo *[152,153]. The strongest genetic evidence implicating TNF in initiation and progression of PD is that a polymorphism (-1031 C) in the TNF promoter that drives transcriptional activity and results in higher than normal TNF production is over-represented in a cohort of Japanese early-onset PD patients compared to late-onset PD patients and unaffected controls [154]. Importantly, this -1031 C polymorphism has been associated with PD risk in an additional study [155]. A second polymorphism in the TNF gene promoter (-308 G/A) that results in elevated serum TNF levels has also been found to be over-represented in early onset sporadic PD [156,157]. Lastly, TNFR1 polymorphisms TNFR1-609 and TNFRI+36 have been found to be significantly decreased in PD patients [156]. In summary, the association of TNF and TNFR1 polymorphisms with PD risk strongly suggests involvement of TNF signaling in PD progression. Meta-analyses will be needed to assess overall genetic association of TNF with PD risk.

Genetic approaches to investigate the role of TNF signaling in the nigrostriatal pathway have involved use of mice deficient in TNF and TNFRs but have yielded conflicting results. In some studies TNFR1 or TNFR2 single-knockout mice or double receptor deficient mice are not protected from MPTP-induced striatal terminal damage or nigral dopaminergic neuron loss [144,158]; whereas in other studies receptor double-knockout mice have less striatal damage than non-transgenic mice [145,159]; and in mice lacking TNF MPTP-induced striatal dysfunction is reduced, but dopaminergic neuron loss is not [146]. Different extents of MPTP lesioning may account for the conflicting results regarding the role of TNF and its receptors in contributing to dopaminergic neuron loss.

Use of non-specific TNF inhibitors including thalidomide, a potent anti-inflammatory and sedative, and minocycline, a tetracycline antibiotic that inhibits TNF synthesis, has also been investigated in MPTP intoxication paradigms in mice and in intranigral LPS rat models of PD with protective outcomes in some [146,160] but not all studies [161]. Due to potential genetic compensation and/or TNF-independent anti-inflammatory effects of these compounds, the direct role of TNF in dopaminergic neuron death could not be established but was certainly inferred. Only recently, a direct role for solTNF signaling in mediating the *in vivo *degeneration of DA neurons in both the 6-OHDA oxidative neurotoxin and chronic LPS models of PD has been demonstrated by the ability of dominant-negative (DN) TNF inhibitors or a lentivirus encoding DN-TNF to attenuate nigral DA neuron loss when delivered intranigrally [162,163]. In summary, combined evidence from histopathologic, epidemiologic, and pharmacologic studies supports a role for TNF in eliciting dopaminergic neuron loss and nigrostriatal degeneration, suggesting TNF neurotoxicity may underlie the progressive loss that occurs in humans with PD.

### TNF in Alzheimer's disease (AD)

The first indication of a contribution for TNF signaling in AD was the presence of TNF at amyloidogenic plaques in post-mortem analysis of AD brains [164]. In pre-clinical studies, transgenic Tg2576 mice which overexpress a mutated form of human amyloid precursor protein (APP) and develop amyloid plaque pathology display elevated TNF levels around fibrillar plaques consistent with findings in human tissue [165-167]. The localization of TNF within plaques in both animal models and human brains prompted investigations into genetic associations between AD and TNF and its receptors. A genome-wide screen found that three TNF polymorphisms (-308, -238 promoter polymorphisms and a 10.5 kb upstream microsatellite TNFa) which result in elevated TNF secretion, formed a haplotype that was associated with AD [168]. However, others have found that these polymorphisms increase the age of AD onset [169]. In addition to genetic linkage with TNF, the genetic association between the TNF receptors (TNFR1 and TNFR2) and AD was investigated. Specifically, the genes for TNFR1 and TNFR2 reside on chromosome 1p and chromosome 12p, respectively and these regions show genetic linkage to late-onset AD; in addition, a genetic polymorphism in exon 6 of the TNFR2 gene was associated with late-onset AD, while no significant association was found between AD and three genetic polymorphisms in the TNFR1 gene [170]. Although these genetic studies implicate TNF and its receptors in modifying AD risk, independent confirmation by additional studies as well as meta-analyses of genetic association studies will be needed to assess the overall genetic effect of these TNF related genes on AD [171,172].

Other evidence of increased inflammation and elevated TNF levels in AD pathology includes the dysregulation of TNF levels and other pro-inflammatory cytokines in AD patients and in transgenic mouse models of AD. Elevated TNF levels appear to correlate with disease progression as higher serum levels of TNF, as well as an increase in the TNF/IL-1β ratio, are present in patients with severe AD compared to individuals with mild-to-moderate disease [76]. However, similar to the case with genetic linkage studies, not all studies found differences in TNF levels between mild and severe disease [76,173]. Transgenic mouse models of AD have provided evidence that inflammation and TNF contribute to disease progression and onset. In three month old mice harboring three familial AD-linked mutations (APP_Swe_, tau_P301L_, and PS1_M146V_) which lead to accumulation of intraneuronal amyloid immunoreactivity in regions that include entorhinal cortex, elevated TNF mRNA levels were reported in the same regions and correlated with the onset of cognitive deficits in these mice [174,175]. Inhibition of solTNF signaling in triple transgenic mice (APP_Swe_, tau_P301L_, and PS1_M146V_) by either hippocampal infusion of DN-TNF inhibitors or intracerebroventricular injection of a lentivirus encoding DN-TNF reduced inflammation-induced accumulation of C-terminal APP fragments in the hippocampus, cortex, and amygdale. In triple (3xTgAD) transgenic mice lacking TNFR1, exposure to chronic systemic inflammation did not result in intraneuronal accumulation of amyloid immunoreactivity, suggesting that solTNF/TNFR1 signaling may be an important regulator of APP processing and that pathological elevation of TNF may contribute to pre-plaque pathology and acceleration of cognitive deficits (McAlpine et al., under review). In support of this idea, the contribution of TNFR1 signaling to AD pathogenesis was recently shown in APP23 transgenic mice which overexpress APP_KM670/671NL_. In these transgenic mice, TNFR1 deletion reduces Aß pathology, microglia activation, BACE1 activity, neuron loss, and memory deficits compared to transgenic APP23 mice expressing normal levels of TNFR1 [176]. Although far from conclusive, a recent report of a single-center, open-label clinical pilot study involving patients with mild-to-severe AD who received semi-weekly perispinal infusion of the anti-TNF biologic etanercept for six months claimed detectable improvement of cognitive performance in patients who received etanercept compared to those that received placebo, raising the interesting possibility that modulation of TNF signaling peripherally or centrally may have effects on cognitive performance in patients with AD [177]. These observations are not surprising in light of published studies that demonstrate TNF is a potent glio-transmitter. However, additional double-blinded placebo-controlled studies will be needed to confirm these promising findings.

### Conclusions and considerations for the future

TNF exerts pleiotropic effects in the CNS and its role in normal brain functions, in particular synaptic scaling, has yet to be fully elucidated. While it is clear that solTNF is toxic to dopaminergic neurons and compromises their survival during chronic inflammatory stimuli in the ventral midbrain, tmTNF and TNFR2 appear to have important roles in myelination and may contribute to hippocampal neurogenesis after ischemic injury. These observations strongly suggest that one potential approach to lessen adverse effects of anti-TNF therapies in the CNS may be to selectively target TNFR1 signaling with localized delivery of inhibitors which spare TNFR2-mediated signaling. As solTNF signals preferentially through TNFR1, selective inhibition of solTNF signaling may be advantageous. This approach may allow efficient neutralization of solTNF in the desired region (i.e. substantia nigra) without eliciting collateral damage to TNFR2/tmTNF-dependent processes in regions of the brain where tmTNF has important homeostatic functions. An additional benefit to selective targeting of solTNF would be sparing tmTNF-dependent functions in the immune system, including proper development and maintenance of immune cell populations as well as self-tolerance and resistance to infectious agents (such as *Mycobacterium *and *Listeria*). Given the growing evidence that TNFR2 may antagonize TNFR1 signaling, a second strategy, which may bestow beneficial effects and allow protection of neuronal populations highly sensitive to solTNF/TNFR1-mediated toxicity, may be to increase TNFR2 expression levels through gene therapy. Lastly, it is important to note that while brain-permeant small molecule inhibitors of TNF, including the immunomodulatory drugs thalidomide and minocycline, may be attractive for CNS applications, their lack of selectivity for solTNF over tmTNF and their inhibitory effects on other inflammatory mediators may contribute to undesirable effects as TNF signaling and other inflammatory cascades would be suppressed globally in the CNS [178].

## Competing interests

The authors declare that they have no competing interests.

## Authors' contributions

The manuscript was written by MKM as part of her doctoral thesis. MGT provided historical perspectives and editorial assistance.
